# Structural Features and Zeolite Stability: A Linearized Equation Approach

**DOI:** 10.1021/acs.cgd.3c00893

**Published:** 2024-01-29

**Authors:** Salvador R.G. Balestra, Noelia Rodríguez-Sánchez, Dayrelis Mena-Torres, A. Rabdel Ruiz-Salvador

**Affiliations:** † Departamento de Sistemas Físicos, Químicos y Naturales, Universidad Pablo de Olavide, Ctra. Utrera km. 1, Sevilla ES-41013, Spain; ⊥ Centro de Nanociencia y Tecnologías Sostenibles (CNATS), Universidad Pablo de Olavide, Ctra. Utrera km. 1, Sevilla ES-41013, Spain; ‡ Departamento de Deporte e Informática, Área de Lenguajes y Sistemas Informáticos, Universidad Pablo de Olavide, Ctra. Utrera km. 1, Sevilla ES-41013, Spain; § EASYTOSEE AGTECH S. L., c/José Delgado Brackenbury 9, Sevilla ES- 41011, Spain

## Abstract

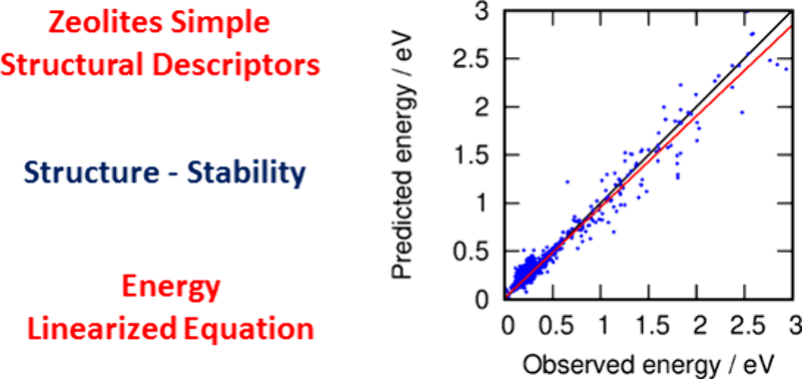

Zeolite stability, in terms of lattice energy, is revisited from a crystal-chemistry point of view. A linearized equation relates the zeolite lattice energy using simple structural data readily available from experiments or modeling. The equation holds for a large range of zeolite energies, up to 3 eV per tetrahedron with respect to quartz, and has been validated internally via two simple machine learning automatic procedures for data fitting/reference partitions and externally using data from recently synthesized zeolites. The approach is certain in locating those recently synthesized zeolites in the energy range of those experimentally known zeolites used in the parametrization of the linearized equation. Hidden intrinsic structural data–energy correlations were found for data sets built from energy-relaxed structures along with energy values computed using the same energy functions employed in the structural relaxation. The asymmetry of the structural features is relevant for an accurate description of the energy.

## Abstract 2

Zeolite stability, in terms of lattice energy, is revisited from a crystal-chemistry point of view. A linearized equation approach is introduced to relate the lattice energy of a wide range of known and hypothetical zeolites and simple structural data. The approach is certain in locating those recently synthesized zeolites in the energy range of those previously known by experiment. The asymmetry of the structural features is relevant for an accurate description of the energy.

## 1 Introduction

Zeolites are crystalline nanoporous solids that are in high demand for industrial, energy, and environmental applications.^1,2^ Molecular confinement within the pore space is responsible for their outstanding catalytic, sorption, separation, and ion-exchange properties.^1,3,4^ The close relationship between the intriguing structure and spectacular performance of these materials has attracted special attention for a deeper understanding of the factors controlling their stability for rational synthesis and the new discovery of zeolites.

One approach to understanding the factors controlling the stability and structure of zeolites is to determine their lattice energy, which is directly related with the formation enthalpy, and it can be readily computed using quantum mechanical based calculations, employing both wave function and density-functional methods.^5−7^ The high accuracy of these calculations is paid for with very high cost. In the second level of cost and accuracy, tight-binding or interatomic-potential-based calculations can be used to access much larger systems in terms of the number of atoms and in real time in the case of molecular dynamics simulations. The latter, although lacking first-principles grounds, can reproduce complex structural features with reasonable accuracy, such as the monoclinic distortion in silicalite MFI and its thermal behavior,^8,9^ the Si–Al distribution in fully and partially ordered zeolite frameworks,^10,11^ structural flexibility upon pressure,^12^ or variation in the nature of extra-framework cations or adsorbate content,^13−15^ including large volume variation by varying the water loading in hydrophilic zeolites,^15,16^ surface structure,^17^ and breathing of pore-delimiting windows under different conditions,^18,19^ among others. It should be noted that the recently developed machine learning potential type of approximation has already reached zeolite science.^20^

Despite the success of quantum mechanics and interatomic potential methods, developing approaches that can describe the energy of zeolites in a much simpler manner has been a matter of interest over the last three decades.^21^ Understanding the stability of zeolites has been a major motivation in these studies by identifying factors that can be used as a measure of their synthesizability. Pioneering work by Akporiaye and Price showed negative linear relations between the coordination sequence and framework density with the lattice energy.^22^ A few years later, the study of the effect of the framework density was extended to a larger set of silica zeolites and AlPOs.^23,24^ The validity of this dependence in MOFs,^25,26^ and in ordered nanoporous metals indicates that the cumulative contributions of the existing interatomic interactions beyond next-neighbors account for this behavior in porous materials.^27^ Zwijnenburg et al. conducted a series of work on the stability of zeolites and related structural factors.^21^ They developed a simple topological model based on polyhedral tiles and the analysis of the face-size distribution that can be used to predict the thermodynamic viability of the synthesis of zeolites built by simple tilings for a fixed average face-size.^13^ Calculation of the tetrahedral distortion in experimentally observed (low-energy) zeolites revealed very little distortion in these zeolites, in contrast to that observed in most hypothetical (high-energy) zeolites.^28^ Sastre and Corma also conducted systematic research on zeolite stability and the predictability of their synthesis. They studied the strain associated with the rings and found that a detailed analysis of the rings is required to understand their effects in a given zeolite framework. Rather than the number of small rings, the number of strained angles in the atoms involving these rings was more important. To illustrate this with representative cases, they showed that double four-membered rings introduce stability in AST, while they are a source of instability in BEC and LTA.^29^ Petrovic et al.^30^ called attention to the role of T-O-T angles in the stability of zeolites, although they observed linear dependence for a few solids, but it was noted that this did not hold for a larger set of zeolites. Further studies have encountered some correlations between local structural parameters, such as T-O and T-T distances and T-O-T angles, and the stability of silica- or aluminophosphate-based zeolites,^31−33^ and similarly in the case of T-T-T angles^34^ and O–O distances.^35^

In recent years, several works have appeared, which use artificial intelligence-based methods for the study of zeolite structure and stability.^20,36−43^ Pioneering work came from Rajagopalan et al., who made some structural data correlations of zeolites using data mining methods,^44^ while Carr et al. applied machine learning data classification tools to identify the topology classes of the structures of zeolites collected in the ISCD.^45^ Ten years later, Helfrecht et al.^39^ took a subset of the PCOD database, which contains zeolites below 30 kJ mol^–1^ per T atom with respect to quartz (∼311 meV, with >300000 structures),^46^ and conducted an interesting study with descriptors based on the Smooth Overlap of Atomic Position representation (SOAP). They concluded that such general descriptors may be better than “classical descriptors” used in zeolite fields (angles, distances, or ring distribution). By analyzing the database using the first three kernel principal components of this descriptor, they captured the essential features of the zeolite data set of Deem et al. According to the authors, these first principal components were strongly correlated to the volume and energy of the structures. With this method, the authors state that they can build a new “atlas” of zeolites based on atomic environments, instead of traditional void spaces or composite building units. Ma et al.^40^ developed a machine learning approach to study the thermodynamic factors associated with the synthesis of zeolites with Al, Si, and P and computed the free energy phase diagram for the formation of ATS-, ATO-, and AFI-type zeolites with different Si:Al:P compositions. Grajciar et al. trained neural network potentials allowing the design of >20k new zeolites with lattice energies in the range of synthesizable zeolites.^20^

One of the most desirable targets behind the study of the connection between structural stability and geometrical descriptors is what is called “feasibility”.^31−34,47^ However, the term ‘(likely) feasible’ is not straightforward. Some zeolites are ‘(likely) unfeasible’ but end up being synthesized through mechanisms not considered in the stated criterion. An example is the novel zeolite ZEO-5,^48^ with a newly stabilized triple-four-membered ring (T4R) using a new synthesis mechanism. The energy criterion seems trivial: the lower the formation enthalpy (once the synthesis method and systems are consistently accounted for), the more feasible the zeolite tends to be. In addition, within a porous medium, the more porous it is, the more difficult it is to stabilize the crystal (i.e., the denser it is, the more stable it is). However, calculating these formation enthalpies is usually not easy. Hence, geometric criteria (that *implicitly* contain relationships with the formation enthalpy) have been developed. These geometric criteria are based on relating some collective variables of zeolites, which typically involve means of distances, angles, or distortions and are typically easy to calculate. For example, Li et al.^32^ published two linear equations that relate angles and distances (TO ∝ TOT and OO ∝ OTO).

In this study, we claim that the conceptual design of new zeolites and a deeper understanding of zeolite stability can be achieved by using a simple algebraic expression that can unify the classical geometrical features (i.e., distances, angles, and distortions) and cell energies of zeolites. Therefore, we focus on developing an equation to explicitly relate the formation energies to these (classical) collective variables. These, were extracted and the energy was estimated using an online available code (github.com/salrodgom/zeolite-analyser). With this aim, we combined the experiences gained thus far to explore whether this would be possible using a simple linearized equation. Note that the main goal here is not to provide the best artificial intelligence-based computational estimation of the energy of zeolites (or even an accurate energy equation for the enormous energy range of considered zeolites), for which nonlinearized methods will obviously be better choices. Although they can provide more accurate predictions of zeolite energies, they are less transparent for mapping the relationship between geometrical features and zeolite energies.

## 2 Methods and Computational Details

To build a general understanding of the correlation between structural features and zeolite lattice energies, it is necessary to work with an overall set of structures that encloses the general features of the zeolite space, in such a manner that it can be taken as a representative collection of structures. Ten sets of zeolite structures were used in this study (Table 1, where *Si* denotes the set, and *i* = 1*–*10). Sets *S1–S4* contain the available 233 noninterrupted frameworks IZA database (URL: iza-structure.org/databases),^49^ differing in the treatment of the data. Sets *S1*, *S3*, and *S4* were subjected to energy minimization using the GULP program,^50,51^ a cutoff distance of 16 Å was used to calculate the short-range interactions in real space, whereas the Ewald summation method was used for the calculation of long-range interactions.^52^ Both the cell parameters and the ionic positions were relaxed for each configuration. A convergence criterion of 0.001 eV Å^–1^ was used for these forces. The Newton–Raphson minimizer with updating the Hessian matrix by the Broyden–Fletcher–Goldfarb–Shanno (BFGS) approximation^53^ was initially used flowed by the RFO minimizer.^54^ In general, this procedure ensures convergence to real minima (*i.e.*, with no imaginary modes, which has been shown to have particular relevance when dealing with zeolitic materials).^8,9,18,23,55^ For *S1*, the well-known Sander, Leslie, and Catlow (SLC) shell-model interatomic potentials were used,^56^ while for sets *S3* and *S4*, the structures were relaxed with the interatomic potentials of Bushuev and Sastre (BS)^47^ and Ramsahye and Bell (RB),^57^ respectively. Using the SLC potential, ∼ 20% of the optimized structures exhibited initially convergence problems owing to the large coupling between the shell particles of oxygen atoms. To achieve full relaxation of these structures, a second optimization was performed, but the positions of the shell particles were reset at a small distance from the core particles. *S2* was taken without further structural relaxation, as these structures are already minimized by the DLS-76 model.^58^ Thus, we have four sets of similar structures at hand, within 2%, but with enough differences to allow a widening of the structural diversity. To correlate their structural features with the lattice energies, the energies for each *S2–S4* structure were recalculated using the SLC potentials by relaxing the atomic shells and keeping the atomic cores and lattice constants frozen.

**Table 1:** Nomenclature and Details of the Sets of Structures and Structural Descriptors Used^a^

set	number of structures	source	IZA topology	optimization
*S1*	233	IZA database	yes	SLC potential
*S2*	233	IZA database	yes	DLS-76
*S3*	233	IZA database	yes	BS potential
*S4*	233	IZA database	yes	RB potential
*S5*	197	FT database	yes	none
*S6*	13	RCSR	not	SLC potential
*S7*	500	FT database	not	none
*S8*	500	FT database	not	SLC potential
*S9*	4361	Deem database	not	SLC potential
*S10*	4211	Deem database	not	SLC potential

descriptor	structural attribute	descriptor	structural attribute
*D1*	FD	*D24 D25 D26*	TOT^2^ TOT_min_^2^ TOT_max_^2^
*D2*	*Q*	*D27 D28 D29*	TT^2^ TT_min_^2^ TT_max_^2^
*D3 D4 D5*	TO T*O*_min_ TO_max_	*D30 D31 D32*	TTT^2^ TTT_min_^2^ TTT_max_^2^
*D6 D7 D8*	OTO OTO_min_ OTO_max_	*D33 D34 D35*	TO^3^ TO_min_^3^ TO_max_^3^
*D9 D10 D11*	TOT TOT_min_ TOT_max_	*D36 D37 D38*	OTO^3^ OTO_min_^3^ OTO_max_^3^
*D12 D13 D14*	TT TT_min_ TT_max_	*D39 D40 D41*	TOT^3^ TOT_min_^3^ TOT_max_^3^
*D15 D16 D17*	TTT TTT_min_ TTT_max_	*D42 D43 D44*	TT^3^ TT_min_^3^ TT_max_^3^
*D18 D19 D20*	TO^2^ TO_min_^2^ TO_max_^2^	*D45 D46 D47*	TTT^3^ TTT_min_^3^ TTT_max_^3^
*D21 D22 D23*	OTO^2^ OTO_min_^2^ OTO_max_^2^		

a FT stands for Foster and Treacy database and RSCR for Reticular Chemistry Structure Resource (rcsr.anu.edu.au/).^60^

Structural set *S5* was taken from a selection of zeolites with IZA topologies that were stored in the Foster and Treacy hypothetical zeolite database (URL: hypotheticalzeolites.net).^59^ A total of 200 zeolites were selected by creating 10 subsets of 20 structures, in which the framework densities were within a range of 1 T site by 1000 Å^3^ (e.g., from equal to or greater than 10 < FD < 11.0 T/1000 Å^3^, and so on until 19 < FD < 20 T/1000 Å^3^). The atomic core positions of these structures were not relaxed to maintain the severe structural distortions, thereby enhancing the diversity of the structural features of the studied zeolites with IZA topologies. The lattice energies of this set were computed in a manner similar to those of *S2*, *S3*, and *S4*.

Sets *S6–S10* were constructed from structures whose topologies are not included in the IZA database. *S6* contains 13 structures taken from the RCSR database,^60^ which are nonporous silicates such as quartz, coesite, tridymite, and cristobalite that share the same primary building units as zeolites (i.e., SiO_4_ tetrahedra). Sets *S7* and *S8* contained the same zeolites but with and without structural relaxation (500 zeolites each) and were obtained from the Foster and Treacy zeolite database with hypothetical frameworks. Therefore, the selection was similar to that for *S5*. However, in this case, subsets of 50 structures were built with 10 < FD < 21 T/1000 Å^3^. Sets *S9* and *S10* were also built using hypothetical zeolite frameworks but taken from a selection of the Deem’s database.^46^*S9* contains structures labeled in the database as 800AAAA and *S10* labeled 833AAAA, where A represents digits from 0 to 9. Sets *S7*, *S9*, and *S10* were subjected to heavy structural relaxation under the same conditions and interatomic potentials as *S1*. The lattice energies of the unrelaxed structures of set *S8* were calculated in the same manner as those for sets *S2–S5*. As can be noted, the SLC potential was selected to compute the energy of the zeolites, considering its capability to reproduce widely diverse structures, including low symmetry configurations, as mentioned above.

We used seven structural features to describe the structure of zeolites. One of them, the framework density (FD), is independent of the local structure (i.e., a global descriptor), whereas the other five are simple structural data: T-O and T-T distances (TO and TT) and the O-T-O, T-O-T, and T-T-T angles (OTO, TOT, and TTT). The seventh is the tetrahedrality coefficient, *Q*, computed according to Zinmmermann et al.^61^ For each structural feature, the average value was considered. As the energy surfaces for atomic displacement are not symmetric (*i.e*., in general, the structural feature distribution is not normal), it is necessary to use more descriptors to account for this. A simple approach involves calculation of the average, minimum, and maximum values for each descriptor and structure. The nomenclature used in this study for each set of structures (*Si*) and descriptors (*Di*) is summarized in Table 1. Because many structural features display near-harmonic behavior close to the structural minima, for each descriptor, except for *D1* and *D2* (FD and *Q*, respectively), the square values were also considered as new numerical attributes. Moreover, the third power of each was considered as a new numerical attribute to account for deviation from harmonicity. A homemade code developed in FORTRAN was used to compute the required structural descriptors and automatically prepare the GULP input files for the cell optimization procedure (github.com/salrodgom/zeolite-analyser).

The general-purpose machine learning Weka program^62^ was used to parametrize the targeted equation. This code uses the standard linear regression method for prediction and applies the Akaike information criterion (AIC)^63^ for model selection, penalizing complex models in favor of simple ones, to avoid overfitting. To validate the internal fitting procedures, cross-validation and percentage split methods were applied. In cross-validation (*k*-fold), the data set was divided into *k* equal parts. In each iteration, *k* – 1 folds were chosen as the training set, and the remaining fold was chosen as the test set. In each iteration, we have one trained model, and the final score is the average of the scores of all of them. We selected ten folds for this study. In the percentage split method, the data set was split randomly into two parts: one for training and one for testing. We selected 70% for the training and 30% for the testing. Preliminary tests with *S1* (IZA relaxed structures) showed a significant correlation between the descriptors, as expected from Li et al.^32^ They also showed that the correlations are likely to disappear when hypothetical structures are considered. Therefore, to avoid automatic deletion of data by Weka, collinear attributes were neither eliminated, nor an a priori attribute selection method was applied. Automatic and nonautomatic attribute selection (e.g., by using a Principal Component Analysis) was further investigated for all considered sets, in the Section S3 in the SI. The data consist of attributes that vary by several orders of magnitude, potentially impacting the Mean Absolute Errors (MAE) of the fitted equations. To mitigate this effect, we regularized each descriptor using the range present in the entire data set of considered zeolite structures (10714 structures). Given that, generally, the distributions are not normal, we employed the difference between the maximum and minimum values of each descriptor for normalization instead of variance. The minimum and maximum values for each descriptor are detailed in Table S1. Cell energy was also regularized using as a reference the cell energy of the quartz (already included in the database) and dividing by the number of T atoms. The used quartz cell energy was −128.70335084 eV/T-atom.

## 3 Results and Discussion

The zeolite frameworks compiled in the IZA database, which are those experimentally observed, constitute the natural starting points for connecting the structural features and the relative stability of zeolites. Table 2 summarizes the goodness of the linear fits (*r* and MAE) for an increasing number of structural descriptors. Because the energy data were computed with the SLC potentials, *S1* has special relevance in this study because it is composed of IZA structures relaxed with this potential. The structurally closed sets were expected to have frameworks near but slightly displaced from the SLC-generated energy minima. The extent to which these departures from the minima associated with the chosen interatomic potentials affect the link between the structural descriptors and energy is an unexplored question. To further evaluate the impact of structural distortion on IZA topologies, *S5* is also included in columns sixth and seventh along with sets *S1–S4*.

**Table 2:**
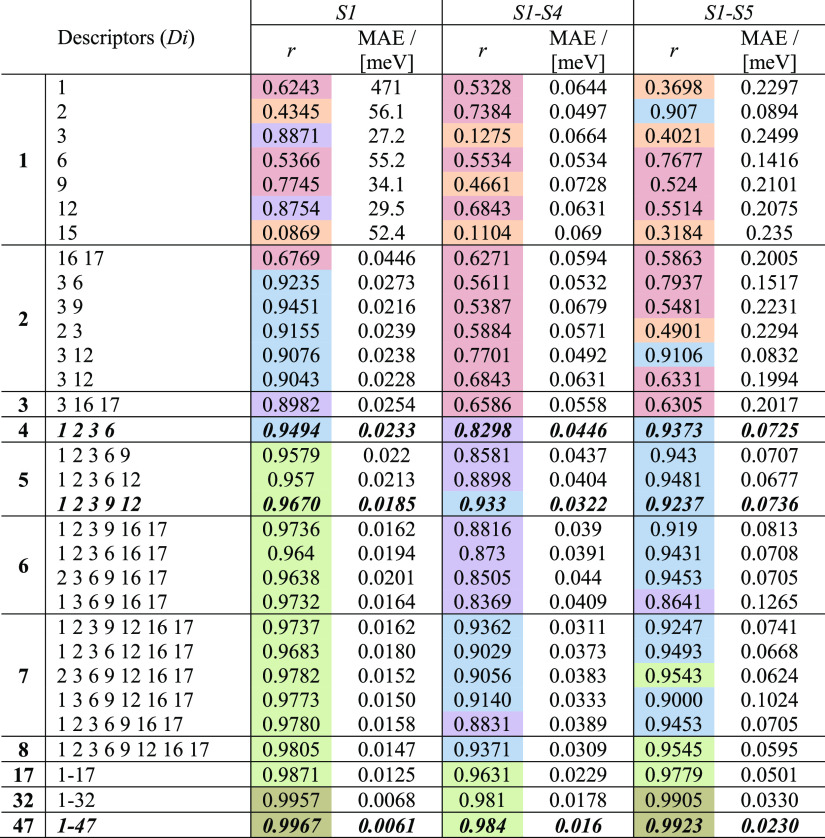
Goodness-of-Fit (*r* and MAE) of the Lattice Energies at Varying Complexities for Zeolites with IZA Topology: *S1* (Columns 3 and 4), *S1–S4* (Columns 5 and 6), and *S1–S5* (Columns 7 and 8)^a^

a The number of descriptors used in each trial is presented in the first column. The descriptor labels and additional nomenclature are listed in Table 1. The cells are colored following this: orange (*r* < 0.5), red (0.5 < *r* < 0.8), purple (0.8 < *r* < 0.9), blue (0.9 < *r* < 0.95), green (0.95 < *r* < 0.99), and olive-green (*r* > 0.99).

An unexpected behavior was observed in five numerical experiments, where only two simple structural descriptors were required to have a Pearson correlation coefficient of *r >* 0.9 when fitting the data of *S1*. This explains the structural correlations observed in IZA relaxed structures by Li et al.^32^ The next experiment explores the effect of small structural differences. In this regard, using these pairs of descriptors in the other two types of experiments for sets *S1–S4* and *S1–S5* resulted in a significantly lower quality of fit. With only four descriptors, we obtained reasonably good fits, with *r* ∼ 0.94, for *S1* and *S1–S5*. While *r* > 0.95 are reached with several configurations of five descriptors, 17 descriptors are needed for such values in sets *S1–S4*. In the case of sets *S1–S5*, it is possible to find one combination of seven and another of eight descriptors with *r* > 0.95, but the MAE values are increased by a factor of 3. Depending on the choice of the seven descriptors, MAE can be increased by a factor of 10, which highlights the importance of the selection. By increasing the number of descriptors was increased to 17, the quality of the fit was close in the three structural sets of configurations, and better agreement was achieved by increasing the number of descriptors to 32 and 47. Nevertheless, differences were still observed in the mean absolute errors, which decreased from left to the right. See the SI for more details and Figures S1a-S1i for fittings using four, five, and 47 descriptors.

The analysis in Table 2 allows us to unify the results reported thus far in the literature,^21,22,24,28,29,31−35,43^ where the use of a limited number of structural descriptors is sufficient to provide a good description of the energy of the zeolite structures that have been previously relaxed by energy minimization or a related method. We conclude that in relaxed structures, there is a hidden intrinsic correlation among structural descriptors that seems to be broken for structures apart from the energy minima. The basis of this point of view is that the crystal packing of solids creates interatomic distances and angles within narrow intervals for a given family of structures. In porous solids such as zeolites, MOFs, and COFs, crystal packing can allow atomic displacement beyond the ideal positions toward the empty space; thus, large variations can appear during deformation. This is the case for *S5*, in which despite the absence of a new topology with respect to sets *S1–S4*, there are significant differences in the bonds and angles. We speculate that this finding can be extrapolated to all solids, including those that are nonporous, when dealing with structural defect sites or surfaces.

**Figure 1:**
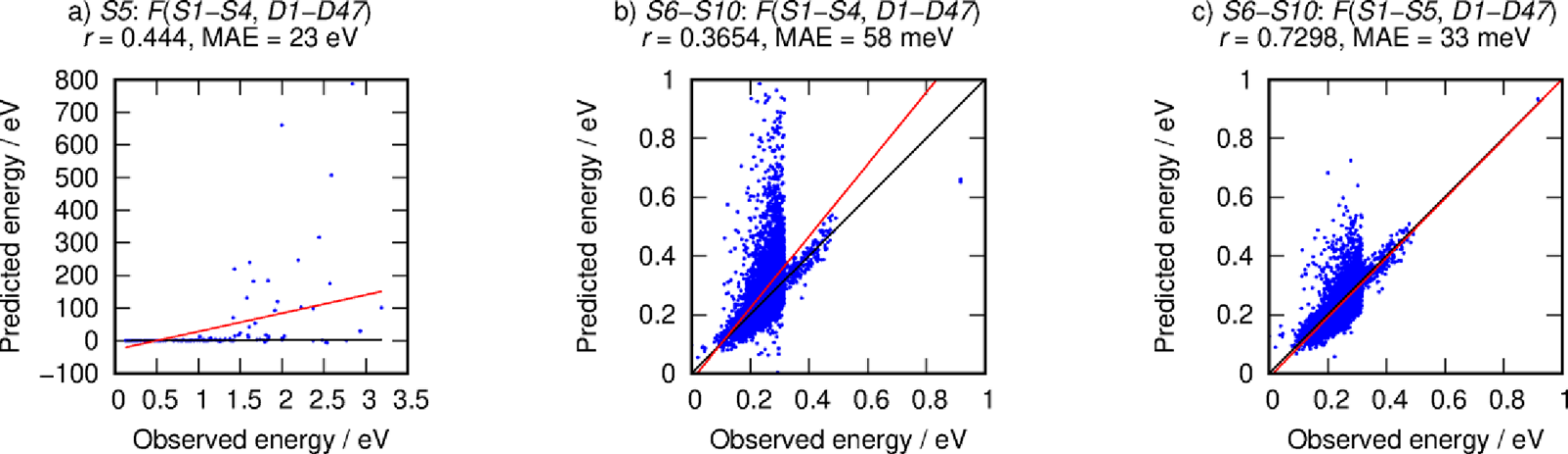
Prediction of the zeolite lattice energies for: (a) deformed IZA *S5* from equation *F*(*S1–S4*; *D1–D47*) (Figure S1h in the SI), (b) non-IZA sets *S6–S10* from equation *F*(*S1–S4*; *D1–D47*), and (c) non-IZA sets *S6–S10* from equation *F*(*S1–S5*; *D1–D47*) (Figure S1i in the SI). The red line is the fitting function, and the black lines are the *y* = *x* curves.

Subset *S5* contained heavily distorted structures. Therefore, it is expected that the linear equation fitted with all of the IZA structures (*S1–S5* sets) would be appropriate for predicting the energy of *S6–S10* non-IZA sets. To help rationalize this, predictions based on fitting to sets *S1–S4* were also included. The predictions of the energy of the unrelaxed distorted IZA *S5* using the linear equation from sets *S1–S4* of the relaxed structures were not accurate and exhibited a large MAE. This is not surprising, as the targeted set had a large number of values of interatomic distances and angles outside the ranges appearing in the training sets, and the *S5* set contained only unrelaxed structures. For example, the range of T*O*_min_ values for sets *S1–S4* is [1.5251:1.6315] Å vs [1.3282:1.6419] Å in *S5*, and similarly for TO*T*_min_ with [114.648:170.616]° vs [90.959:158.984]°. These wide ranges of structural descriptors, resulting from the incorporation of *S5*, covered the interatomic distances and angles of non-IZA sets *S6–S10*. From Figure 1, it is apparent that the structural complexity of the non-IZA hypothetical zeolites of sets *S6–S10* is greater than that found in the IZA topologies, which suggests that a proper description of their energy would also require consideration in the training set non-IZA structures.

**Figure 2:**
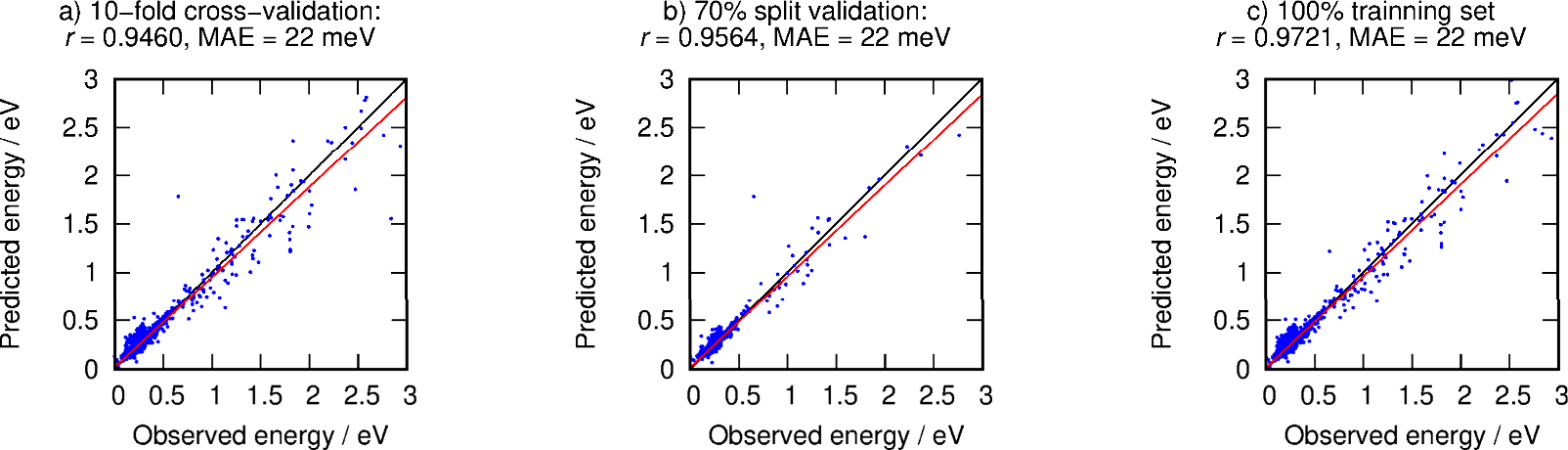
Fitting for lattice energies per T atom of the overall subsets and descriptors, *F*_*1*_ = *F*(*S1–S10*, *D1–D47*), using (a) cross-validation method with ten-fold (plotting 100% data), (b) validation via percentage split at the 70% level (in the panel, we plotted 30% of data), and (c) overall fitting with all structures in the training set *S1–S10* (plotting 100%).

When incorporating non-IZA structures into the training set to fit the zeolite energies of the entire data set (*S1–S10*), the picture was qualitatively different from the case shown in Figure 1, where it was not considered. Figure 2c shows very good results with *r =* 0.9721 and MAE = 0.0214 eV. One can note that the energy range covered here (up to 3 eV/T above quartz) is at least five times larger than those used in previous works. To validate the consistency of this fit and to ensure its value as a predictive crystal engineering tool and gauge the structural stability connection, cross-validation and percentage split methods were used. Both *r* and MSE computed in the two validation procedures were of good quality (0.9460 and 0.0221 eV, respectively, for cross-validation and 0.9564 and 0.0221 eV for 70% split, respectively) and provided confidence in the linearized equation joining simple structural features and zeolite energies. The *r* values of the validation steps echo the heterogeneity of the full set of structures, where, on one hand, there are a variety of experimentally observed topologies (*S1–S5*) and hypothetical frameworks, and on the other hand, there are relaxed and unrelaxed structures. The values of the linear equation coefficients obtained for all the zeolites in the training set are listed in Table S2 (SI). We recall that the goal of our study is not to provide the best possible method for computing zeolite lattice energies as a function of simple structural parameters, but to provide a tool for the direct mapping of their connections. It is worth noting that the energies of the experimentally observed zeolites (*S1*) are typically smaller than 0.3 eV/T, with some exceptions that can reach 1 eV/T, as it occurs in RWY. This is chalchogenide zeolite can be considered as isoreticular to SOD, where the tetrahedra are replaced by supertetradra. Isoreticular or decorated frameworks have been expanded to RHO analogous,^64^ which can be also applied with other topologies and thus opening the way to prepare new zeolites with high energies (ca. 1 eV/T). Experimental evidence so far obtained suggests that energies notably above that of RWY are not expected in synthesizable zeolites, and thus such structures having energies above 2 eV/T are likely to be rather strongly distorted than fully relaxed zeolites with synthesizable opportunity.

Once it is shown that a linearized equation can describe the energy of zeolites using simple structural descriptors, it is possible to conduct numerical experiments by varying the complexity of the fit by selectively omitting some descriptors, as described in Table 3. In this way, it is possible to extract information regarding their relevance in determining the structure–stability relationship. By ignoring one descriptor, including its quadratic and cubic terms, the quality of the fit decreased significantly to *r =* 0.928 for the OTO angles, in contrast to TTT, which had a negligible effect, whereas in the other five cases, the decrease was small (*r* = 0.955). The OTO angle behaves equally with a high impact when it is omitted along with another descriptor, and the TO and TT pairs have similar effects that resemble the OTO angles. One striking observation from the table is that by considering only the average values, a large departure is obtained from the optimal fit, which calls attention to the relevance of the asymmetry of the structural attributes (local diversions from the global descriptor). The absence of cubic terms slightly deteriorates the *r-*coefficient and MAE value; however, the fit is worse for high-energy configurations suggesting large departure from harmonicity at high deformations. Adding the absence of quadratic terms is more impactful in *r* and MEA as well as in the prediction of the energy of high-energy zeolites.

**Table 3:** Goodness-of-Fit (*r* and MAE) of Zeolite Fitting Numerical Experiments at Varying Complexities for All Sets Together (*S1*–*S10*)^a^

exp.	*r*	MAE/[meV]	details	exp.	*r*	MAE/[meV]	details
1	0.9721	21.4	all parameters	13	0.9142	33.1	no OTO and TT
2	0.9595	24.5	no Q	14	0.9593	25.0	no TO and TOT
3	0.9537	29.8	no FD	15	0.9601	24.8	no TO and TTT
4	0.9283	29.0	no OTO	16	0.9235	30.0	no TO and TT
5	0.9616	24.2	no TO	17	0.9685	23.1	no TOT and TTT
6	0.9696	22.6	no TOT	18	0.9412	28.5	no TOT and TT
7	0.9709	21.8	no TTT	19	0.9581	26.8	no TTT and TT
8	0.9598	25.8	no TT	20	0.9211	27.3	AVO
9	0.9423	31.5	no Q FD	21	0.8893	31.1	AVO and no Q
10	0.9003	34.6	no OTO and TO	22	0.8822	37.8	AVO and no FD
11	0.9239	30.1	no OTO and TOT	23	0.9694	22.4	no cubic
12	0.9250	29.4	no OTO and TTT	24	0.9422	30.4	no quadratic and cubic

a The parameters omitted in the experiment are listed in detail, and the AVO represents only the average values.

**Figure 3:**
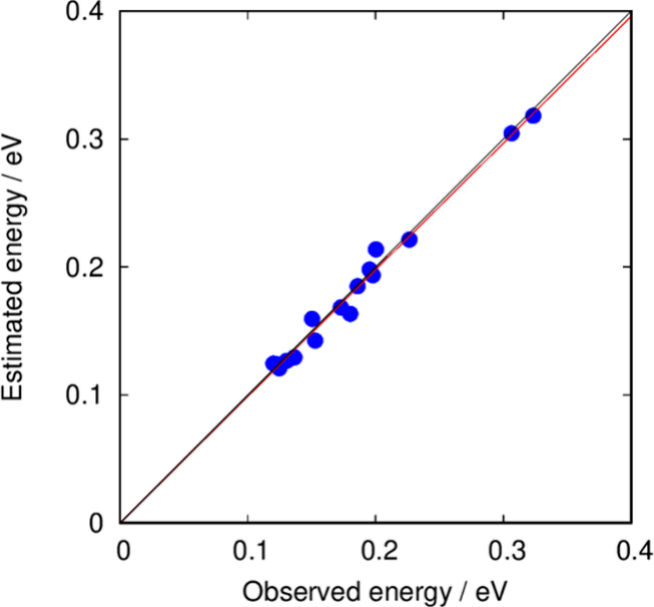
Lattice energies of recent synthesized zeolites as predicted by the reduced parameter and space linearized equation.

The main goal of this study was to focus on the crystal chemistry of zeolites and its effect on stability rather than providing the best description of the lattice energy using a machine learning approach. Nevertheless, it has been shown above that the fitted linearized equation that we have developed can compute, with respect to that computed by SCL potentials, the lattice energy of zeolites within an MAE comparable to the thermal energy. Therefore, it can be used as a tool for the fast calculation of energy. Having in mind that the equation holds for an energy interval that is about 10 times larger than that of most experimental structures and also its linearized form, it would be thus desirable that it could be useful to identify synthesizable zeolites and also highly distorted ones. To demonstrate its capability to identify synthesizable zeolites, we collected a number of the structures of recently reported zeolites (Table 4), not included in the parametrization of the linearized function. The structures were subjected to full lattice energy minimization using the GULP code in the same manner as that for the zeolites in *S1*. The fourth column of Table 4 shows that indeed, the linearized equation provides energies that are compatible with experimental zeolites, and thus, it serves as an external validation of the approach developed in this study. Despite the good qualitative description, one can note relative errors up to ca. 30%, which suggest that fine predictions of the relative stability among synthesizable zeolites is not possible. To improve this capability, within a linearized form of the energy in line with the main goal of this work, it will be needed to reduce both the energy range and the number of parameters, to reduce possible of over fitting effects. Since reducing parameters is a nonstraightforward procedure and it is not a core goal of this work, in the Supporting Information is it presented in detail. The fifth column of Table 4 and Figure 3 show how the predictions can be improved by considering in the parametrization only a subset of structures satisfying both an energetic and structural constrains, as detailed in Section S4 of the Supporting Information. Of note, the MAE is reduced three times to 8 meV.

**Table 4:** Lattice Energies, in meV per T-atom with Respect to Quartz, of a Collection of Recently Synthesized Zeolites Computed by Lattice Energy Minimization with the SLC Potentials, *E*_*obs*_, and Our Estimations Using the Linearized Equations *F*_*1*_ and *F*_*2*_^a^

**name**	IZA code	*E*_obs_/[meV]	*E*_est_ (F_1_)/[meV]	*E*_est_ (F_2_)/[meV]	|Δ*E*|/*E* [%]	ref.
EMM-25	EWF	124.6	157.8	120.1	3	(65)
ZEO-1	JZO	195.3	239.7	198.0	1	(66)
PST-35	PTF	186.0	201.7	184.9	0.5	(67)
PST-29	PWN	180.1	206.6	163.5	10	(68)
ZSM-25	MWF	172.8	239.7	168.5	2	(64)
ZEO-3		226.5	270.3	221.4	2	(69)
ZEO-5		323.4	368.4	318.2	2	(48)
COE-11		150.3	200.3	159.5	6	(70)
ECNU-13a		136.5	161.6	129.4	5	(71)
ECNU-13b		152.3	170.7	142.5	7	(71)
ECNU-23a		130.4	167.6	126.7	3	(72)
ECNU-23b		120.0	165.9	124.5	4	(72)
GAM-3		306.3	306.0	304.4	0.6	(73)
ITQ-69		200.3	221.6	213.8	6	(74)
NUD-3		197.8	218.6	193.6	2	(75)

a The absolute relative errors are displayed for function *F2.*

## 4 Conclusions

The complex relationship between structural features and zeolite stability is revisited here, where it has been shown that some simple structural descriptors are sufficient to describe the lattice energy of zeolites in a simple manner through a linearized equation. Although we do not discuss new routes for the synthesis of zeolites in this study, the following approach allows the mapping of the structure–stability relationship in zeolites and is expected to contribute to the development of new zeolites. The OTO angles appear as structural motifs that have a greater impact on directing the energy landscape of zeolites followed by the TT and TO distances. The influence of the TTT angle is much less decisive in determining the relative energy of zeolites. The inclusion of asymmetry in the values of each descriptor emerged as a very important point, expressed by the minimum and maximum values, along with the averages.

The results indicate a hidden correlation between structural parameters and energy within sets of energy optimized structures. This correlation was established using the same energy gauge employed to correlate the structure and energy. This finding has methodological importance in understanding the relationships between structure and stability and in the application of machine learning in this domain. The previous research has shown that a few descriptors are sufficient to reveal energy trends in experimentally known zeolites, often taken from the IZA database or low-energy selections from hypothetical zeolite databases. By exclusively concentrating on a customized function designed for likely feasible zeolites (or potential future IZA structures), modeling is simplified, energy prediction accuracy is improved, and complexity is reduced by reducing dimensionality. Automatic or manual attribute selection or dimensionality reduction did not produce satisfactory results for the *S1*–*S10* structure set. This highlights the intricate relationship between geometric descriptors and the structural energy. However, specific descriptors such as Q, TO, TT, and TTT are the most representative bases for energy modeling.

For practical applications, we provide two linearized equations that can be used to compute the energies of experimentally determined or hypothetical zeolites. This method uses only simple structural descriptors that are readily available from experiments or modeling approaches. This estimation of the lattice energy using the linearized equation and the required structural descriptors can be easily calculated using the provided code. This is expected to contribute to the design of zeolites and large-scale screening. The wide range equation can be used for any structure, and if it is identified as a synthesizable zeolite, then the reduced range equation can be used to refine the calculation of its energy.

## Supporting Information Available

Structural data in normalized format, with minimum and maximum value for each descriptor; description of the procedures for automatic and nonautomatic attribute selection; discussion about feasibility of zeolite frameworks; table containing coefficients of the linearized equation for computing the lattice energy from structural descriptors (PDF)
Database with the structural and energy data (ZIP)
