# Supplementary material for: Structural Features and Zeolite Stability: A Linearized Equation Approach
Source: Cryst Growth Des. 2024 Jan 29;24(3):938–46. doi: 10.1021/acs.cgd.3c00893 (PMC10853909; doi:10.1021/acs.cgd.3c00893)
Supplement: Supplementary file 1 — cg3c00893_si_001.pdf [file cg3c00893_si_001.pdf]

Supplementary information for “Structural Features and Zeolite Stability: A Linearized Equation Approach”

Salvador R.G. Balestra,[†⊥] *Noelia Rodríguez-Sánchez,*[†] *Dayrelis Mena-Torres,*[‡§] * *and A. Rabdel Ruiz-Salvador*[†⊥] *

[†] Departamento de Sistemas Físicos, Químicos y Naturales, Universidad Pablo de Olavide, Ctra. Utrera km. 1, ES-41013, Sevilla, Spain.

[⊥] Centro de Nanociencia y Tecnologías Sostenibles (CNATS), Universidad Pablo de Olavide, Carretera de Utrera km. 1, Seville, E-41013, Spain

[‡] Escuela Politécnica Superior, Universidad Pablo de Olavide, Ctra. Utrera km. 1, ES-41013, Sevilla, Spain.

[§] EASYTOSEE AGTECH S. L., c/ José Delgado Brackenbury 9, 41011, Sevilla, Spain

Corresponding Authors

A. R. Ruiz-Salvador rruisal@upo.es, ORCID 0000-0002-2004-687X

D. Mena-Torres dmentor@upo.es, ORCID 0000-0001-8610-5791

Authors

S. R. G. Balestra, ORCID 0000-0002-2163-2782

N. Rodríguez-Sánchez, ORCID 0009-0005-9019-835X

Section S1. Mathematical expression and fitted coefficients

Table S1. Data ranges used for descriptor normalization.

Descriptor type	$\overline{\min(D_i)}$	$\overline{\max(D_i)}$	$\min(\min D_i)$	$\min(\max D_i)$	$\max(\min D_i)$	$\max(\max D_i)$
D1	7.7461	37.9034	-	-	-	-
D2	0.2100	1.0000	-	-	-	-
D3-D5	1.5742	1.6660	1.3282	1.6548	1.5744	1.8713
D6-D8	105.9392	109.9492	63.5395	109.4616	109.4808	163.6060
D9-D11	123.9087	175.7053	90.9591	170.6157	124.6023	180.0000
D12-D14	2.8329	3.2351	2.2673	3.2072	2.9105	3.6102
D15-D17	101.5302	112.7831	48.74126	108.6046	109.9063	180.0000

Table S2. Coefficients k_i of the linearized equation (F_1) for the lattice energies of zeolites. The equation has the following expression: $E_{est} = D_0 + \sum_{i=1}^{47} k_i D_i$. The descriptor are regularized using the max. and min. values of **Table** S1.

Parameter, D_i	Coefficient, k_i	Parameter, D_i	Coefficient, k_i	Parameter, D_i	Coefficient, k_i
D_1, **FD**	-0.3741	D_2, Q	-0.7575	D_0	4.9554
D_3, $\overline{\mathrm{TO}}$	0.3717	D_{18}, $\overline{\mathrm{TO}}^2$	-0.8941	D_{33}, $\overline{\mathrm{TO}}^3$	1.5727
D_4, $\mathrm{TO}_{\min}$	-2.0333	D_{19}, $\mathrm{TO}^2_{\min}$	0.5799	D_{34}, $\mathrm{TO}^3_{\min}$	0.4708
D_5, $\mathrm{TO}_{\max}$	0.6529	D_{20}, $\mathrm{TO}^2_{\max}$	-2.0339	D_{35}, $\mathrm{TO}^3_{\max}$	1.8244
D_6, $\overline{\mathrm{OTO}}$	0.4955	D_{21}, $\overline{\mathrm{OTO}}^2$	-5.2814	D_{36}, $\overline{\mathrm{OTO}}^3$	3.1999
D_7, $\mathrm{OTO}_{\min}$	-0.1377	D_{22}, $\mathrm{OTO}^2_{\min}$	0.5487	D_{37}, $\mathrm{OTO}^3_{\min}$	-0.2603
D_8, $\mathrm{OTO}_{\max}$	-0.4629	D_{23}, $\mathrm{OTO}^2_{\max}$	1.5215	D_{38}, $\mathrm{OTO}^3_{\max}$	-0.8001
D_9, $\overline{\mathrm{TOT}}$	2.0877	D_{24}, $\overline{\mathrm{TOT}}^2$	-1.9925	D_{39}, $\overline{\mathrm{TOT}}^3$	0.7249
D_{10}, $\mathrm{TOT}_{\min}$	0.3608	D_{25}, $\mathrm{TOT}^2_{\min}$	-0.0377	D_{40}, $\mathrm{TOT}^3_{\min}$	-0.1869
D_{11}, $\mathrm{TOT}_{\max}$	0.4396	D_{26}, $\mathrm{TOT}^2_{\max}$	-0.6033	D_{41}, $\mathrm{TOT}^3_{\max}$	0.2432
D_{12}, $\overline{\mathrm{TT}}$	-3.5034	D_{27}, $\overline{\mathrm{TT}}^2$	2.1687	D_{42}, $\overline{\mathrm{TT}}^3$	-0.1351

D$\boldsymbol{D_{13}}$, $\mathbf{TT_{min}}$	-3.5413	D_{28}, TT^2_{min}	3.5786	D_{43}, TT^3_{min}	-1.1826
$\boldsymbol{D_{14}}$, $\mathbf{TT_{max}}$	-0.4083	D_{29}, TT^2_{max}	0.7027	D_{44}, TT^3_{max}	-0.1132
$\boldsymbol{D_{15}}$, $\overline{\mathbf{TTT}}$	0.3632	D_{30}, $\overline{TTT}^2$	-0.7074	D_{45}, $\overline{TTT}^3$	0.4935
$\boldsymbol{D_{16}}$, $\mathbf{TTT_{min}}$	-0.5293	D_{31}, TTT^2_{min}	1.1257	D_{46}, TTT^3_{min}	-0.7438
$\boldsymbol{D_{17}}$, $\mathbf{TTT_{max}}$	0.0459	D_{32}, TTT^2_{max}	-0.0536	D_{47}, TTT^3_{max}	0.0475

Section S2: Automatic and non-automatic attribute selection

To minimize overfitting caused by high correlation among certain attributes in the dataset, an experimentation was conducted to reduce the dimensionality and collinearity of the problem's attributes. Four procedures were considered for this purpose. **Table S3** summarizes the main results of the experimentation using the dataset of all zeolites (*S1-S10*). A 10-fold cross-validation approach was employed.

The conducted procedures were as follows:

1) Modifying the input parameters of the algorithm provided by the ***weka.classifiers.functions.LinearRegression*** function, available in the Weka automated learning environment.[1] Specifically, adjustments were made to the ***AttributeSelectionMethod*** and ***EliminateColinearAtributes*** parameters. The ***weka.classifiers.functions.LinearRegression*** function employs linear regression for prediction and utilizes the Akaike Information Criterion (AIC),[2] for model selection.

   The following input parameters were varied within this function:

   a) ***AttributeSelectionMethod:*** Setting used to select attributes for linear regression, comprising:
      i) no attribute selection,
      ii) attribute selection using M5's method (iterative attribute removal based on the smallest standardized coefficient until no improvement is observed in the error estimate given by the AIC), and
      iii) a greedy selection using the Akaike information metric.

   b) ***EliminateColinearAttributes***, aimed at removing collinear attributes.

   The ***NumDecimalPlaces*** parameter was set to 10 in all experiments. The remaining parameters (***DoNotCheckCapabilities***, ***OutputAdditionalStats***, ***Minimal***, ***BatchSize***, ***Debug***, and ***Ridge***) were used with their default values. By resetting the ***AttributeSelectionMethod*** parameter to ***Greedy method*** and ***EliminateColinearAttributes*** to 'True' (without performing any manual reduction), the dataset was reduced to 36 attributes, resulting in a correlation of $r = 0.9455$ and a MAE of 23.8 meV. This represented the best-case scenario observed, as no substantial improvement was observed.

2) Perform a Principal Component Analysis (PCA) to transform the initial dataset. The dataset is preprocessed using the filter ***weka.filters.unsupervised.attribute.PrincipalComponents*** available in Weka. The default parameters remain unchanged. Dimensionality reduction is achieved by selecting enough eigenvectors to represent 95% of the variance in the original data. However, the obtained results decrease the correlation and increase the error. In some cases, they do not reduce the dimensionality but rather transform it, complicating the equation for energy calculation, as it would require computing the components beforehand.

3) Apply attribute selection algorithms using correlation-based evaluation methods. Two attribute selection models are applied beforehand. The ***CfsSubsetEval***,[3] evaluation method is employed, utilizing the ***PSOSearch***,[4] and ***BestFirst*** algorithms:

   a) ***PSOSearch***: It navigates the attribute space using the Particle Swarm Optimization (PSO) algorithm.
   b) ***BestFirst***: It explores attribute subsets using a greedy hill-climbing approach enhanced with a backtracking capability. It controls the level of backtracking allowed by determining the number of consecutive non-improving nodes. ***BestFirst*** can start with an empty attribute set and search forward, start with the full attribute set and search backward, or initiate from any point and search in both directions. In this context, the results obtained reduced correlation and increased error. However, the application of the ***PSOSearch*** algorithm substantially reduced the search space to just 11 attributes, maintaining a correlation of $r = 0.8906$ and an MAE of 39.1 meV. The relevant attributes obtained are: OTOangleave, TOdistmin, TTdistave, OTOangleave2, TOdistmin2, TOdistmax2, OTOangleave3, OTOanglemax3, TOdistmax3, TTdistmax3, and Q.

4) Manual elimination. From a crystallographic standpoint, the powers of 2 and 3 of the descriptors T-T and T-T-T, as well as the powers of 3 for T-O, O-T-O, and T-O-T, are removed due to their lower weight compared to others. Combined with attribute selection using the Greedy method and removal of collinear attributes from the function, the search space is reduced to 23 attributes, resulting in a correlation of $r = 0.9555$ and an MAE of 26.2 meV. The relevant attributes after this combination include: FDensity, OTOanglemin, OTOangleave, OTOanglemax, TOdistmin, TOdistave, TOdistmax, TOTangleave, TOTanglemax,

TTTanglemin, TTTangleave, TTTanglemax, TTdistmin, TTdistave, TTdistmax, OTOanglemin2, OTOangleave2, OTOanglemax2, TOdistmin2, TOdistave2, TOdistmax2, TOTanglemin2, and Q.

Table S3. Attribute selection contribution to the fitness.

	weka.classifiers.functions.LinearRegression - parameters		Results		
**Reduction case**	**attributeSelectionMethod**	**eliminateColinearAtributes**	**No. Att**	**Correlation coefficient**	**MAE**
Variation of function parameters	no attribute selection	False	47	0.946	0.0221
	no attribute selection	True	43	0.945	0.0238
	M5 method	False	43	0.9459	0.0221
	M5 method	True	41	0.9451	0.0238
	Greedy method	False	42	0.9464	0.0221
	Greedy method	True	36	0.9455	0.0238
Application of the pre-prosessing method: Principal Components	no attribute selection	False	47 (13 dimensions)	0.8823	0.0371
	M5 method	True	37 (12 dimensions)	0.8822	0.0371
	Greedy method	True	37 (12 dimensions)	0.8822	0.0371
Implementation of the Attribute Selection Method: PSOSearch	no attribute selection	False	11	0.8906	0.0391
Implementation of the Attribute Selection Method: BestFirst	no attribute selection	False	8	0.8604	0.0414
Manual reduction of attributes of powers 2 and 3 of T-T and T-T-T descriptors and powers 3 of T-O, O-T-O and T-O-T	no attribute selection	True	26	0.9554	0.0262
	M5 method	True	24	0.9554	0.0262
	Greedy method	True	23	0.9555	0.0262

Section S3: (Extra) manually attribute selection

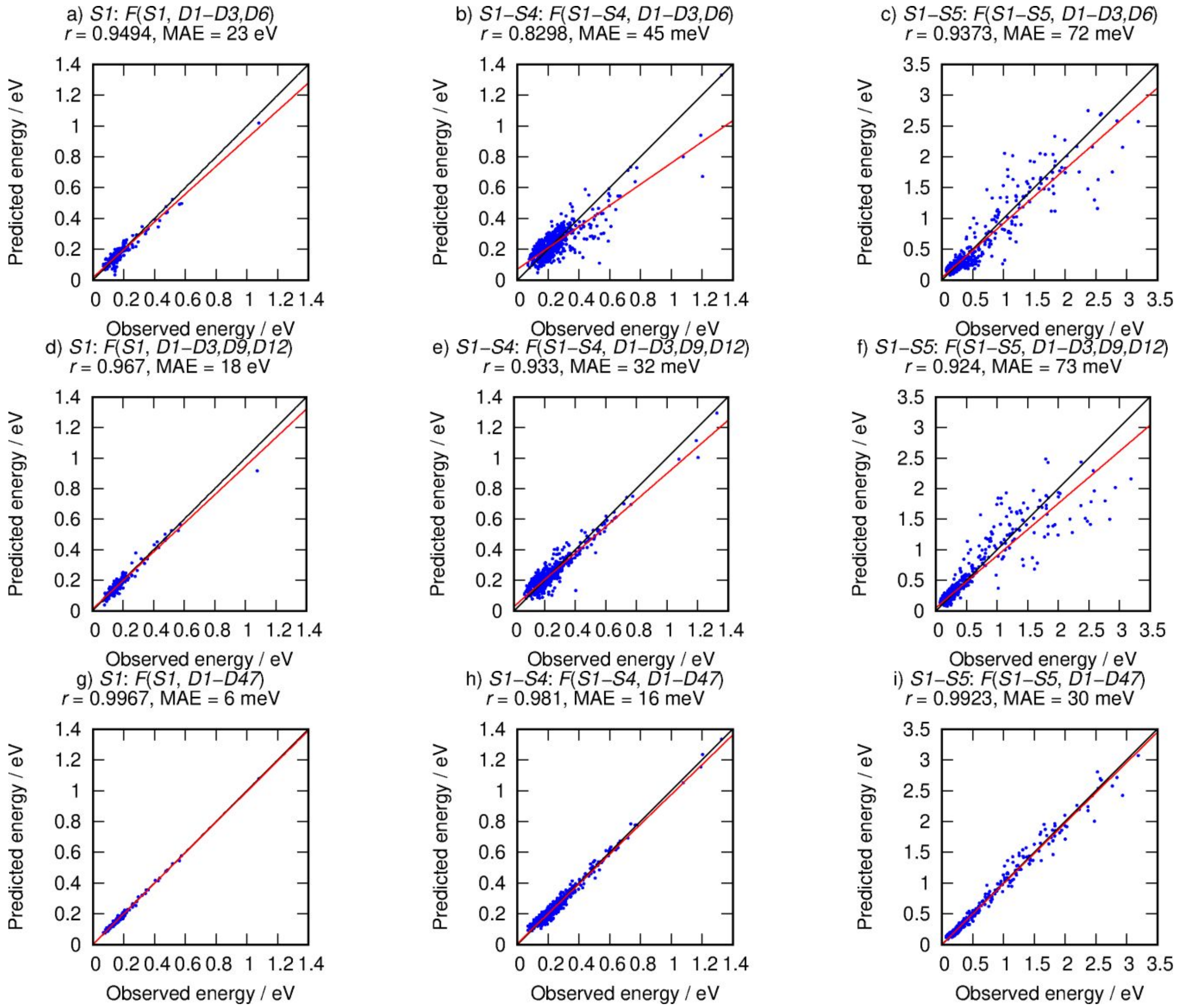

Figure S1. Energy fit of IZA zeolites on increasing complexity of the linearized equation

Section S4: About "feasibility"

To achieve a linear function between energy and geometrical descriptors within a narrower energy range thereby increasing the accuracy of the fit, we can limit its application range to synthesizable zeolites. However, this concept is not easily straightforward (as discussed in the main text), thus necessitating the need to make certain assumptions.

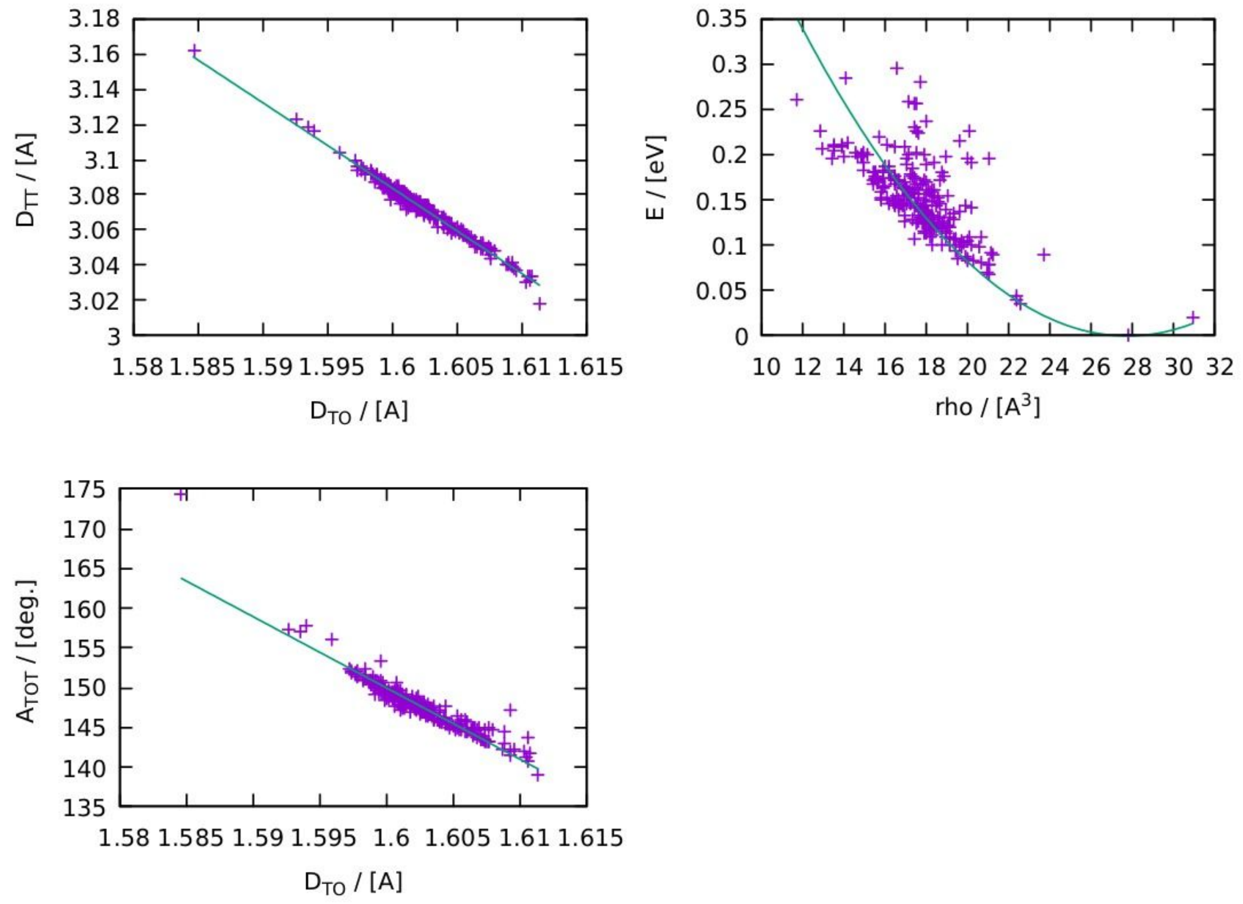

Figure S2. TT-TO (top-left), TOT-TO, and E-FD plots for synthetized zeolites (excluding set *S11*) and dense polymorphs (set *S6*).

We define, the energetic feasibility function as $\varphi_{E_i} = |E_i - f(FD_i)|$, as function of the cell energy E for a zeolite i and the equation $f = f(FD)$, show in the **Figure S2** (top-right) in solid green. In the same way, we defined the TT-feasibility equation as $\varphi_{\mathrm{TT}_i} = |\overline{\mathrm{TT}}_i - g(\overline{\mathrm{TO}}_i)|$, and

TOT-feasibility as $\varphi_{\mathrm{TOT}_i} = \left|\overline{\mathrm{TOT}}_i - h(\overline{\mathrm{TO}}_i)\right|$ (solid green lines in **Figure S2 top-left**, and **bottom-left**, respectively. The functions f, g, and h are, respectively:

$$f(FD_i) = k_f(FD_i - FD^0)^2,$$

with $k_f = 1.36529029359175$ T eV/Å^3, and $FD^0 = 27.735093$ Å^3 / (1000 T),

$$g(\overline{\mathrm{TO}}_i) = k_g(\overline{\mathrm{TO}}_i - \mathrm{TO}^0),$$

with $k_g = 4.84556092406946$ [-], and $\mathrm{TO}^0 = 2.23634925310887$ Å, and

$$h(\overline{\mathrm{TO}}_i) = k_g(\overline{\mathrm{TO}}_i - \mathrm{TO}^1),$$

with $k_h = 897.933499699582$ deg Å^{-1}, and $\mathrm{TO}^1 = 1.766982908$ Å.

One we have defined the feasibility functions (φ_{E_i}, φ_{TT_i}, and φ_{TOT_i}) for each i zeolite, we applied the following filter to the entire database (excluding the *S11*, the new synthesized zeolites) to obtain the ensemble of likely feasible zeolites (~1800 zeolites): $\varphi_{E_i} < 0.095$ eV, $\varphi_{\mathrm{TT}_i} < 0.003$ Å., and $\varphi_{\mathrm{TOT}_i} < 3$ deg. The filter thresholds were chosen so that the set of synthesized zeolites (including sets *S1*, *S6* and *S11*) are "feasible". For the filtered subset of zeolites, we subsequently applied the fitting procedure of the linearized equation (r = 0.97 and MAE of 8 meV). We obtained a performance score of $r = 0.96$ and MAE of 8 meV (for the S11, see **Figure 3**).

Bibliography

(1) Eibe Frank; Mark A. Hall; Ian H. Witten. Data Mining: Practical Machine Learning Tools and Techniques. In *The WEKA Workbench*; Morgan Kaufmann, Fourth Edition, 2016.

(2) Akaike, H. A New Look at the Statistical Model Identification. *IEEE Trans Automat Contr* **1974**, *19* (6), 716–723. https://doi.org/10.1109/TAC.1974.1100705.

(3) Hall, M. A. Correlation-Based Feature Subset Selection for Machine Learning. *Thesis submitted in partial fulfilment of the requirements of the degree of Doctor of Philosophy at the University of Waikato* **1988**.

(4) Moraglio, A.; Di Chio, C.; Poli, R. Geometric Particle Swarm Optimisation; 2007; pp 125–136. https://doi.org/10.1007/978-3-540-71605-1_12.
